# Evaluation of the clinical use of *MGMT* methylation in extracellular vesicle-based liquid biopsy as a tool for glioblastoma patient management

**DOI:** 10.1038/s41598-024-62061-8

**Published:** 2024-05-18

**Authors:** Rocío Rosas-Alonso, Julian Colmenarejo-Fernández, Olga Pernía, Miranda Burdiel, Carlos Rodríguez-Antolín, Itsaso Losantos-García, Tania Rubio, Rocío Moreno-Velasco, Isabel Esteban-Rodríguez, Virginia Martínez-Marín, Paloma Yubero, Nicolas Costa-Fraga, Angel Díaz-Lagares, Rafael López-López, Eva Díaz-Martin, Juan F. García, Catalina Vivancos Sánchez, Maria Luisa Gandía-González, Gema Moreno-Bueno, Javier de Castro, Inmaculada Ibánez de Cáceres

**Affiliations:** 1 Cancer Epigenetics Laboratory, INGEMM, La Paz University Hospital, Paseo La Castellana 261, Edificio Bloque Quirúrgico Planta-2, 28046 Madrid, Spain; 2 Biomarkers and Experimental Therapeutics in Cancer, IdiPAZ, Madrid, Spain; 3 Biostatistics Unit, IdiPaz, Madrid, Spain; 4 Department of Pathology, La Paz University Hospital, Madrid, Spain; 5 Department of Medical Oncology, La Paz University Hospital, Madrid, Spain; 6 Cancer Epigenomics Laboratory, Epigenomics Unit, Translational Medical Oncology Group (ONCOMET), IDIS, University Clinical Hospital of Santiago (CHUS/SERGAS), Santiago de Compostela, Spain; 7 Centro de Investigación Biomédica en Red de Cáncer (CIBERONC), Instituto de Salud Carlos III, Madrid, Spain; 8 Department of Medical Oncology, University Hospital Complex of Santiago de Compostela, Santiago de Compostela, Spain; 9 MD Anderson International Foundation, Madrid, Spain; 10 Department of Pathology, MD Anderson Cancer Center, Madrid, Spain; 11 Department of Neurosurgery, La Paz University Hospital, Madrid, Spain; 12 Departamento de Bioquímica, Universidad Autónoma de Madrid (UAM), Instituto de Investigaciones Biomédicas ‘Alberto Sols’ (CSIC-UAM), IdiPAZ, Madrid, Spain

**Keywords:** Small extracellular vesicles (sEVs), MGMT, Methylation, Glioblastoma, Liquid biopsy, Biological techniques, Cancer, Genetics, Biomarkers

## Abstract

Glioblastoma (GB) is a devastating tumor of the central nervous system characterized by a poor prognosis. One of the best-established predictive biomarker in IDH-wildtype GB is O6-methylguanine-DNA methyltransferase (MGMT) methylation (mMGMT), which is associated with improved treatment response and survival. However, current efforts to monitor GB patients through mMGMT detection have proven unsuccessful. Small extracellular vesicles (sEVs) hold potential as a key element that could revolutionize clinical practice by offering new possibilities for liquid biopsy. This study aimed to determine the utility of sEV-based liquid biopsy as a predictive biomarker and disease monitoring tool in patients with IDH-wildtype GB. Our findings show consistent results with tissue-based analysis, achieving a remarkable sensitivity of 85.7% for detecting mMGMT in liquid biopsy, the highest reported to date. Moreover, we suggested that liquid biopsy assessment of sEV-DNA could be a powerful tool for monitoring disease progression in IDH-wildtype GB patients. This study highlights the critical significance of overcoming molecular underdetection, which can lead to missed treatment opportunities and misdiagnoses, possibly resulting in ineffective therapies. The outcomes of our research significantly contribute to the field of sEV-DNA-based liquid biopsy, providing valuable insights into tumor tissue heterogeneity and establishing it as a promising tool for detecting GB biomarkers. These results have substantial implications for advancing predictive and therapeutic approaches in the context of GB and warrant further exploration and validation in clinical settings.

## Introduction

Treatment of central nervous system (CNS) tumors is a significant unmet medical need. According to the WHO 2016 Classification of gliomas, IDH wild-type glioblastoma (GB) is the most common CNS tumor and has an inherently poor prognosis^1^. The WHO CNS tumor classification of 2016 was updated in 2021^2^. Conventional treatment of GB consists of maximum surgical tumor resection, followed by radiotherapy with concomitant temozolomide (TMZ) and adjuvant TMZ according to the STUPP protocol^3^. GB is the most aggressive glioma, and despite advances in surgical techniques and antitumoural treatments, patients have a median overall survival (OS) of 15 months^4^.

Currently, one of the best-established predictive biomarker in IDH-wildtype GB is O^6^-methylguanine-DNA methyltransferase (*MGMT*) gene promoter methylation, which is associated with TMZ response and better survival^4–6^. The 2-year survival rate is 12% in patients harboring unmethylated *MGMT* tumors compared to 49% in methylated *MGMT* (m*MGMT*) tumors^4^. The cytotoxic actions of TMZ have been associated with its ability to form DNA adducts that cause tumor cell death. The repair process in tumor cells is carried out by the MGMT protein, so an unmethylated *MGMT* gene promoter, leading to high protein expression, is associated with resistance to TMZ treatment. However, the m*MGMT* gene promoter is associated with the silencing of MGMT expression and thus the inability of the cell to maintain the repair process^5,7^.

The tumor *MGMT* methylation status is routinely obtained from surgical resection or biopsy in clinical practice. However, due to tumor location or tumor necrosis, it is sometimes difficult to obtain enough tissue for biomarker testing^8^. Tumors are known to release markers into the peripheral blood in various ways; thus, liquid biopsy is emerging as an alternative tool that could overcome the boundaries associated with tumor sampling^9,10^. Circulating tumor DNA (ctDNA), small extracellular vesicles (sEVs) and circulating tumor cells are being used to detect and monitor cancer biomarkers^10,11^ but are heavily influenced by the type, stage, and location of the tumor^12^. However, detecting m*MGMT* in blood remains a challenge for patients with glioma because the amount of ctDNA is reduced, making it necessary to identify new approaches that increase the sensitivity of liquid biopsy in these patients^12–15^. A sEV-based liquid biopsy approach may provide new insights in this regard, as *IDH* mutations have been detected in the sEV DNA of patients with GB^16^. Therefore, this study aimed to determine the utility of sEV-based liquid biopsy to analyze m*MGMT* status as a predictive biomarker and disease monitoring tool in patients with glioblastoma.

## Methods

### Study design and patients

We conducted a prospective exploratory study at Hospital La Paz (Madrid, Spain) between January 2017 and March 2021. 50 patients were included in the study. Eligibility criteria included patients with histopathological confirmation of *IDH* wild-type GB according to WHO 2016 Classification, age ≥ 18 years, signed informed consent and an Eastern Cooperative Oncology Group (ECOG) performance status of 0 to 3^17^ and first-line STUPP treatment candidates. Once patients had progressed on the STUPP protocol, further treatment decisions were left to the choice of the prescribing physician. In addition, we included eight healthy donors in the study from volunteers at our center. Eligibility criteria for healthy donors were absence of oncological disease at the time of sample collection, ECOG 0, age ≥ 18 years and signed informed consent. The exclusion criteria for both groups included not meeting the inclusion criteria or failing to sign the informed consent form. The primary endpoint was to assess the sensitivity and specificity of sEV-DNA m*MGMT* with respect to tumor methylation results. The association between sEV-DNA m*MGMT* and OS was also evaluated, as well as its usefulness in patient follow-up according to MRI images. The MRI response was interpreted based on the modified criteria for radiographic response assessment recommendations proposed by Ellingson et al.^18^. Surgical complete resection was defined based on absence of tumor contrast enhancement on early postoperative MRI (< 72 h from surgery). This study was approved by the institutional Ethics Committee (PI-2887) according to the Helsinki Declaration.

### Specimen characteristics

Blood samples were obtained in EDTAK2 BD Vacutainer tubes (Becton Dickinson, USA) after surgery and before starting the concomitant treatment with STUPP protocol. Namely, between 3 and 9 weeks after surgery, depending on each patient's recovery time. Plasma was obtained by a first centrifugation at 2500*g* for 10 min at 4 °C. The supernatant was transferred to a new tube followed by a second step of centrifugation at 35,000*g* for 20 min at 4 °C. Plasma samples were stored in 1.5 ml aliquots at − 80 °C until further processing.

### Assay methods

#### sEV isolation

Ultracentrifugation (UC) is the most common method used for sEV isolation^19^. Between 1–1.5 ml of plasma was thawed and filtered through a 0.2 µm polyether sulfone membrane (Fisher Scientific, Massachusetts, USA) to eliminate larger particles. Samples were diluted in filtrated phosphate buffered saline (PBS) up to a volume of 7 ml to reduce viscosity and increase the yield of sEV isolation^20^. Then, the PBS-diluted plasma was filled into 13.5 ml thick-walled polycarbonate reusable tubes specific for UC processing (Beckman Coulter, USA). Samples were ultracentrifuged at 100,000×*g* for 70 min at 4 °C using a fixed-angle rotor (Rotor 70.1 Ti, K factor of 36) in an Optima L-100 XP ultracentrifuge (Beckman Coulter, USA). The pellet was washed with 7 ml of filtrated PBS and ultracentrifuged again under the same conditions. The pellet containing the sEVs was suspended in 150 μl of filtered PBS.

#### sEV characterization

Two independent methods, nanoparticle tracking analysis (NTA) and transmission electron microscopy (TEM), were used on a subset of 18 samples to characterize sEVs as recommended^19^.

Nanosight LM10 (Malvern, UK) employs NTA technology that enables precise analysis of the size distribution and concentration of all types of suspended nanoparticles. From 10 μl of the sEV suspension obtained after UC, a 1:100 dilution was carried out with filtered PBS to achieve the concentration range recommended by the manufacturer (10^8^–10^9^ particles/ml). This dilution was injected into the device, and two 60-s videos for each sample were generated and analyzed by NTA 3.0 software, providing particle size and distribution data.

The sEV morphology was confirmed in a few samples by TEM (n = 4). Fifty microliters of sEV suspension obtained after UC was fixed with 4% paraformaldehyde (PFA) (pH 7.2). Five microliters of this suspension was placed on parafilm and a formvar/carbon grid. The grid was washed out with PBS and then fixed with 1% glutaraldehyde. Once the sEVs were fixed on the grid, they were stained with uranil-oxalate, embedded in methylcellulose-uranil and visualized with TEM (JEOL JEM 1010, Japan) at 100 kV and 100,000×. Digital Micrograph software was used for image visualization and processing.

#### Nucleic acid isolation and *MGMT* methylation analysis

DNA from formalin-fixed and paraffin-embedded (FFPE) tissue samples was processed in routine clinical practice. Samples were fixed in 10% formalin and subsequently embedded in paraffin within the pathology department. Hematoxylin and eosin staining was performed on each sample to confirm the presence of tumor nuclei exceeding 20%. The FFPE samples were stored at room temperature until DNA isolation. FFPE tissue samples were deparaffinised using xylene. DNA extraction from tissue samples followed established protocols using phenol–chloroform and chloroform, culminating in suspension in 20 μl of Tris–EDTA buffer solution (TE 1×), as previously described^7^. sEV-DNA was isolated by organic extraction (phenol:chloroform) according to standard protocols and finally resuspended in TE 1×^21^.

For m*MGMT* analysis, sEV-DNA was modified using sodium bisulfite according to standard protocols^22^. Next, nested PCR was performed. The reaction mixture contained 6 μl of bisulfite-modified DNA, 3 μl of buffer (Biotools Buffer 10×), 4 μl of dNTPs (10 mM), 1 μl of MgCl_2_ (50 nM), 2 μl of primers (100 ng/µL) and 0.75 μl of enzyme (Biotools DNA polymerase 1 U/µl). The nested PCR conditions were 94 °C for 5 min, followed by 40 cycles at 94 °C for 1 min, 54 °C for 1 min, 72 °C for 1 min and ending with 1 cycle at 72 °C for 8 min. The product of the reaction was a 166 bp amplicon. The sequences (5′–3′) used were as follows: GGATATGTTGGGATAGTT (forward primer) and CCTACAAAACCACTCRAAACT (reverse primer). Six microliters of the product resulting from this PCR was used as a template for quantitative methylation-specific PCR (qMSP). The analysis of qMSP in tumors was performed according to the previously published formula^7^, while the analysis of qMSP in sEV-DNA was performed according to the formula log(−∆Ct). Supplementary Fig. 2 shows a summary of all primers used.

### Statistical analysis

*mMGMT* was recorded as a continuous variable, but in oncology, it is common to convert it into a categorical form by using cutoff points^23^. The primary endpoint was to assess the sensitivity and specificity of sEV-DNA m*MGMT* in relation to tumor methylation results. To categorize the m*MGMT* results obtained in tumor tissue, we used the cutoff point established and validated in our previous study^7^. For sEV-DNA samples, the quantification of methylated tumor DNA is challenging in blood samples due to its low abundance, typically estimated to be less than 1%. To overcome this limitation, we employed the log(−∆Ct) formula, where any positive ∆Ct value was considered a methylated result to minimize false negatives reported in liquid biopsy^24^. This allows us to detect methylated DNA when the FAM probe initiates amplification, even if it occurs significantly later than the amplification of the VIC probe (representing unmethylated DNA) as expected. This approach enables the accurate quantification of tumor DNA methylation levels in blood samples with higher sensitivity and precision.

Concordance between dichotomous variables was studied using sensitivity, specificity, positive predictive value (PPV), negative predictive value (NPV) and the percentage of observed agreements (concordance percentage) with their respective 95% confidence intervals (95% CI). The kappa index has also been analyzed with its respective significance, and it is considered a more robust method because it takes into account coincidences that occur by chance^25^. Kappa index result was interpreted as follows: values ≤ 0 as indicating no agreement and 0.01–0.20 as none to slight, 0.21–0.40 as fair, 0.41–0.60 as moderate, 0.61–0.80 as substantial, and 0.81–1.00 as almost perfect agreement^26^. The association between m*MGMT* and clinicopathological status (qualitative variables) was analyzed by the chi-squared test (surgical resection extension, ECOG) or Fisher's exact test (sex). For the comparison between qualitative m*MGMT* (qualitative) and age (quantitative data), Student's t test was used for independent data. The normality of these variables was assessed using the Kolmogorov–Smirnov test. The OS analysis was performed using Kaplan‒Meier tests. A *p* value less than 0.05 was considered statistically significant. Given that the extension of resection has been previously reported to have an impact on OS^27–30^, a subgroup analysis of OS was performed among those patients who underwent incomplete resection. The statistical analysis was carried out using R (version 4.0.2) and survival, KMsurv and survminer packages.

### Ethics approval and consent to participate

This study was conducted under the approval of the ethics committee of the La Paz University Hospital with the ethics number PI-2887. The study was performed according to the Helsinki Declaration. Informed consent was signed by all patients.

## Results

### Patient characteristics

**Figure 1:**
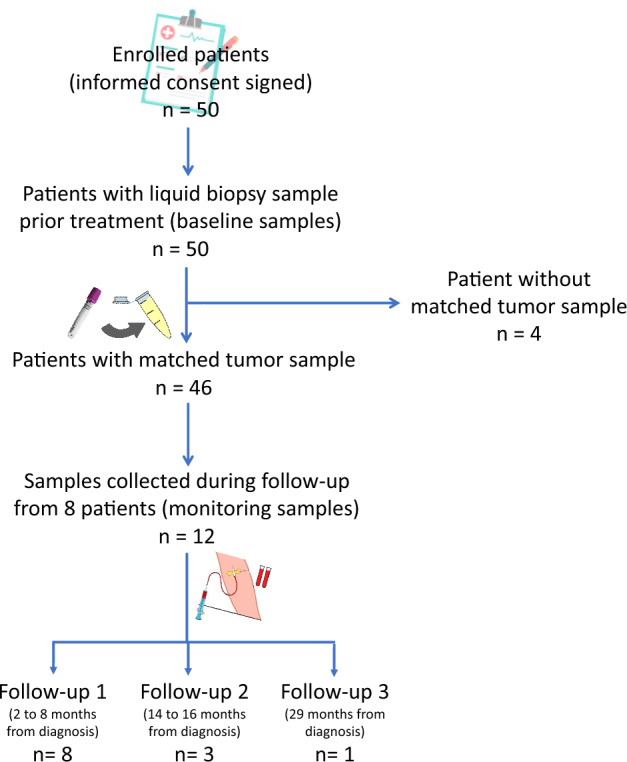
Diagram of patients included in the study and collected samples. The general characteristics of the patients are shown in Table 1, while the methylation results derived from the paired samples are shown in Table 2.

From January 2017 to March 2021, 50 patients were included in the study, with a median follow-up time of 13 months. Three patients had an insufficient tumor cell percentage in the sample (< 20%), and one patient was diagnosed at another hospital, so the tumor was not available to perform MGMT studies. The blood sample collection flow chart and the clinical and demographic characteristics of the patients are shown in Fig. 1 and Table 1, respectively.

**Table 1:** Baseline demographic data and clinical characteristics of the patients.

Characteristics	N = 50
Age (mean, SD, min and max)	
All patients	61 ± 12 (25–81)
Female	59 ± 10 (39–76)
Male	62 ± 13 (25–81)
Gender (n, %)	
Female	21 (42%)
Male	29 (58%)
Extent of resection (n, %)	
Complete resection	27 (54%)
Subtotal resection	16 (32%)
Biopsy only	7 (14%)
ECOG performance status (n, %)	
0	23 (46%)
1	19 (38%)
2	7 (14%)
3	1 (2%)
Tumor *MGMT* methylation status (n, %)	
Methylated	19 (38%)
Unmethylated	27 (54%)
Not available	4 (5%)

*SD* standard deviation.

### Extracellular vesicle characterization

**Figure 2:**
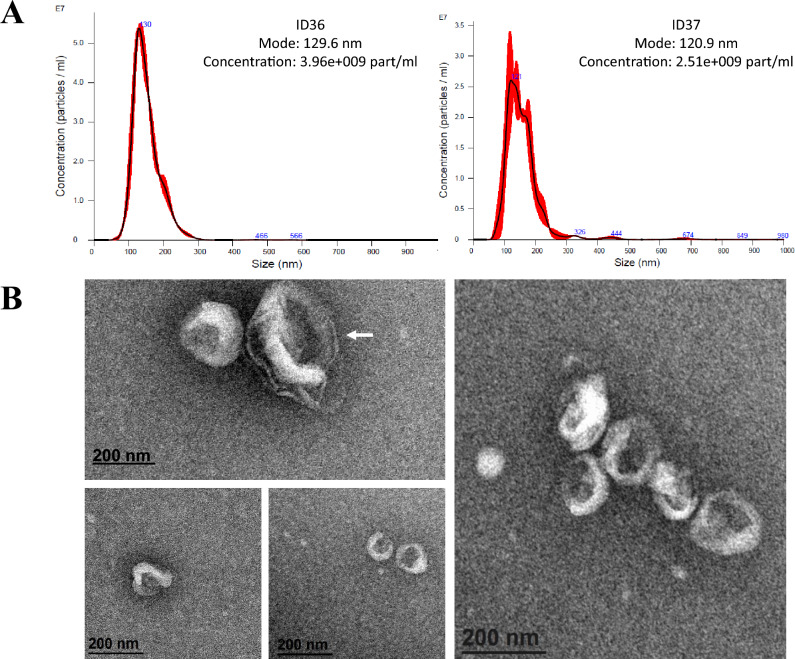
Extracellular vesicle characterization. (**A**) Visualisation of circulating sEVs obtained from the plasma of GB patients using NTA. Examples of extracellular vesicle size distribution profiles in four out the fourteen samples obtained by NTA. NTA confirms the presence of extracellular vesicles below 150 nm. ID36 and ID37 are used to identify patients according to Table 2. (**B**) Visualisation of circulating sEVs obtained from the plasma of GB patients using transmission electron microscopy (EM). Images were taken at a magnification of 120,000. EM images showed small vesicles of approximately 120–130 nm in diameter, clustered in some cases. The arrow indicates a larger vesicle (> 200 nm).

NTA analysis was performed on those samples where there was sufficient plasma to perform both determinations, MGMT methylation and NTA assays. We also confirmed the presence of sEVs by EM. The use of both methods confirmed that most of the EVs observed were less than 200 nm in size and had the characteristic cup shape of sEVs (Fig. 2).

### Evaluation of m*MGMT* in liquid biopsy using sEV-DNA is consistent with the biomarker analysis in tumor tissue DNA

As expected, mMGMT methylation was not detected in the sEV DNA of any of the eight healthy donors (Supplementary Fig. 1).

The tissue sample was available for 46 out of the 50 patients for whom blood was available. *MGMT* promoter methylation was detected in 19 (41.3%) of these patients using DNA of tumor tissue origin and in 14 (28%) patients using sEV-DNA. There were seven cases (14%) in which the presence of methylation was detected in the tumor DNA but not in the matching sEV DNA samples. In contrast, there were two patients in whom m*MGMT* was detected in sEV-DNA but not in tumor tissue-DNA (Table 2).

**Table 2:** *MGMT* methylation status in samples analysed from tissue and from sEVs in paired samples from glioblastoma patients and clinical data of the patients. M: *MGMT* methylated, U: *MGMT* unmethylated

Sample ID	Sex	Age	sEVs-DNA *MGMT* methylation (baseline)	Tumor *MGMT* methylation	Tumor *MGMT* methylation percentage (%)	Extent of resection	Follow-up (months)	Patient status at last contact	Number of treatment lines*
1	M	25	M	M	78.0	Biopsy	66.1	Alive	1
2	M	69	M	M	81.6	Complete resection	17.8	Exitus	2 (fotemustine)
3	M	63	M	M	7.1	Complete resection	26.5	Exitus	2 (fotemustine)
4	M	79	M	M	100.0	Biopsy	3.7	Alive	1
5	F	49	U	U	0.0	Complete resection	7.6	Exitus	2 (fotemustine)
6	M	57	U	U	0.0	Complete resection	16.3	Exitus	1
7	M	61	U	U	0.0	Incomplete resection	50.4	Exitus	1
8	F	65	U	U	0.0	Incomplete resection	9.5	Exitus	1
9	F	70	U	U	0.0	Incomplete resection	1.2	Exitus	1
10	M	48	U	U	0.0	Complete resection	24.0	Exitus	2 (fotemustine)
11	M	34	U	Not available	Not available	Complete resection	29.6	Exitus	2 (irinotecan)
12	M	69	U	U	0.0	Complete resection	19.4	Exitus	1
13	F	65	U	U	0.0	Complete resection	1.5	Exitus	1
14	F	54	M	U	0.0	Biopsy	11.1	Alive	1
15	F	47	U	M	94.8	Complete resection	13.4	Alive	1
16	M	47	U	U	0.0	Complete resection	12.9	Alive	2 (lomustine + bevacizumab)
17	M	63	U	U	0.0	Complete resection	17.1	Exitus	2 (fotemustine)
18	F	39	U	U	0.0	Incomplete resection	18.6	Exitus	1
19	M	77	U	U	0.0	Complete resection	11.3	Alive	2 (fotemustine)
20	M	69	U	Not available	Not available	Biopsy	1.8	Exitus	1
21	F	43	U	M	99.5	Complete resection	4.6	Alive	1
22	M	50	U	M	41.9	Complete resection	16.9	Exitus	1
23	M	60	U	U	0.0	Complete resection	38.1	Exitus	1
24	M	61	U	U	0.0	Complete resection	10.6	Exitus	1
25	F	61	U	U	0.0	Incomplete resection	11.2	Exitus	1
26	F	70	U	U	0.0	Biopsy	18.1	Exitus	1
27	M	65	U	U	0.0	Complete resection	7.4	Exitus	2 (fotemustine)
28	M	68	M	M	87.7	Incomplete resection	34.2	Alive	1
29	F	66	M	M	7.1	Biopsy	4.0	Exitus	1
30	F	56	U	U	0.0	Complete resection	3.6	Alive	1
31	F	59	M	M	72.2	Incomplete resection	9.8	Exitus	1
32	F	73	M	M	47.2	Incomplete resection	10.7	Exitus	1
33	M	68	U	U	0.0	Incomplete resection	1.6	Exitus	1
34	F	51	M	M	79.4	Complete resection	6.9	Alive	1
35	F	51	U	U	0.0	Incomplete resection	45.2	Exitus	2 (temozolamide)
36	M	69	U	U	0.0	Incomplete resection	2.4	Exitus	1
37	M	61	U	U	0.0	Complete resection	13.1	Exitus	1
38	F	70	M	M	92.7	Incomplete resection	18.1	Alive	1
39	F	50	M	Not available	Not available	Incomplete resection	5.7	Exitus	1
40	M	71	M	U	0.0	Complete resection	9.7	Exitus	1
41	M	39	U	U	0.0	Incomplete resection	8.2	Exitus	2 (fotemustine)
42	F	76	U	U	0.0	Complete resection	17.7	Alive	1
43	M	81	U	U	0.0	Incomplete resection	8.4	Exitus	1
44	M	78	U	M	10.1	Complete resection	22.8	Alive	1
45	M	76	U	M	69.1	Complete resection	14.2	Alive	1
46	M	71	U	M	24.5	Complete resection	19.6	Alive	1
47	F	57	M	M	100.0	Complete resection	19.4	Alive	1
48	M	60	M	M	100.0	Complete resection	15.4	Exitus	2 (bevacizumab)
49	F	67	U	Not available	Not available	Biopsy	11.2	Alive	1
50	M	61	U	M	97.7	Incomplete resection	15.7	Exitus	1

*In all cases, the patient received first-line treatment according to the STUPP protocol. The second line of treatment is given in brackets.

Of the first seven cases, six corresponded to patients who had undergone macroscopically complete resection as described intraoperatively by the neurosurgeon. Complete resection was confirmed in two of these patients (ID22 and ID44) by early postoperative magnetic resonance imaging (MRI). Although the targeted surgical lesion was completely resected for patient ID21, this is a case of multifocal GB, where a known residual lesion was confirmed by postoperative MRI. The extent of resection could not be confirmed by early postoperative MRI for patient ID45, given that images were not assessable due to the patient’s movements when the images were taken. Last, although intraoperative complete resection was described for patient ID46, tumor remnants were observed in the early postoperative MRI. Postsurgical MRI was not available for the final patient.

The concordance percentage obtained between sEV-DNA and tumor tissue-DNA was 80.4% (95% CI: 66.1–90.6). The sensitivity to detect methylation in sEV-DNA according to the methylation observed in tumor tissue-DNA was 63.2% (95% CI: 38.4–83.7), while the specificity was 92.6% (95% CI: 74.7–99.1). The PPV and NPV were 85.7% (95% CI: 60.2–95.9) and 78.1% (95% CI: 62.2–86.7), respectively (Table 2). The kappa index showed moderate concordance (k = 0.580) between tumor tissue-DNA and sEV-DNA (p < 0.001). If we only considered the subgroup of patients who underwent incomplete resection or biopsy, we achieved a concordance of 90.0% (95% CI: 68.3–98.8), a sensitivity to detect methylation in sEV-DNA of 87.5% (95% CI: 47.3–99.7) and a specificity of 91.7% (95% CI: 61.5–99.8), with a PPV of 87.5% (95% CI: 51.3–97.9) and an NPV of 91.7% (95% CI: 63.6–98.6). The kappa index was 0.792 (substantial concordance) between tumor tissue-DNA in the subgroup of patients who underwent incomplete resection or biopsy and sEV-DNA (p < 0.001).

### Determination of m*MGMT* status in sEV-DNA as a survival predictor biomarker

**Figure 3:**
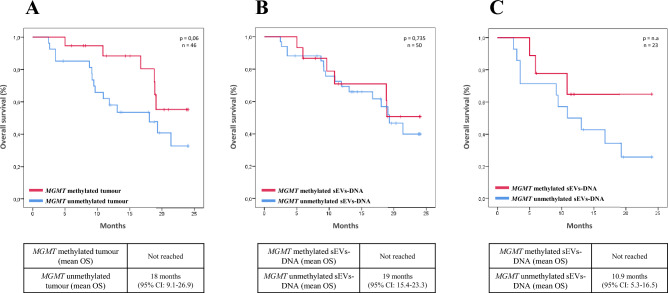
Overall Survival curves in our patient cohort. (**A**) Kaplan–Meier estimates of overall survival according to *MGMT* methylation in tumor sample. (**B**) Kaplan–Meier estimates of overall survival according to *MGMT* methylation in sEVs-DNA. (**C**) Kaplan–Meier estimates of overall survival according to *MGMT* methylation in sEVs-DNA in the subgroup of patients without complete resection. *n.a.* not available.

In our patient cohort, mMGMT tumor status discriminated patients with respect to OS without reaching statistical significance (p = 0.06). After a median follow-up of 24 months, the median OS in patients with *MGMT*-unmethylated tumors was 18 months (95% CI: 9.1–26.9), while the median OS was not reached in patients with *MGMT*-methylated tumors after 24 months of follow-up (Fig. 3A). When overall survival was analyzed with respect to the *MGMT* methylation status in the DNA of the sEVs, regardless of the extent of resection, no significant differences were observed (p = 0.735) (Fig. 3B).

This result led us to evaluate the median OS in the group of patients who did not undergo complete resection, in which a poorer OS was achieved in patients with *MGMT*-unmethylated versus *MGMT-*methylated sEVs-DNA (10.9 months; 95% CI: 5.3–16.5 versus not reached (p value not assessable due to lack of events) (Fig. 3C).

### *mMGMT* in sEV-DNA as a patient monitoring tool

**Figure 4:**
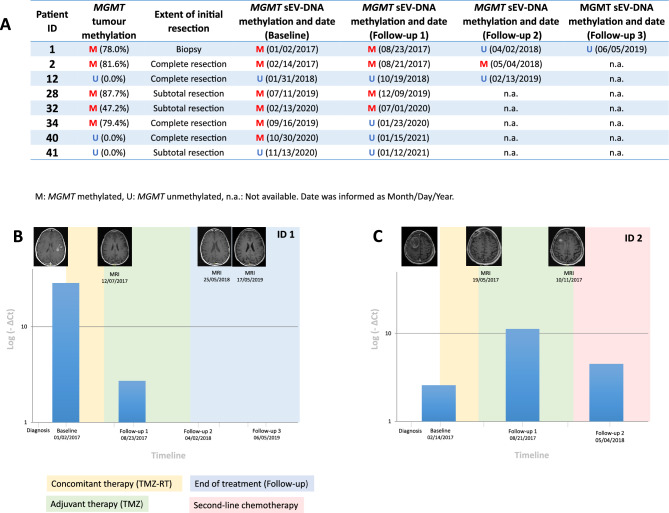
Serial monitoring sEVs-DNA methylation status. (**A**) sEVs-DNA methylation status of all samples from the 8 monitored patients. (**B**) Changes in *MGMT* methylation ∆Ct levels detected in sEVs-DNA-based liquid biopsy in patients ID1. (**C**) Changes in *MGMT* methylation ∆Ct levels detected in sEVs-DNA-based liquid biopsy in patients ID2.

To study the implications of measuring m*MGMT* status in blood along the course of the disease, we collected 20 baseline and follow-up samples from eight patients (Fig. 4A).

Patients ID1, ID2, ID28, ID32 and ID34 had an *MGMT* methylated tumor. Patient ID1 was diagnosed with an unresectable m*MGMT* tumor. sEV-DNA at baseline showed m*MGMT*, which decreased over time during treatment and was undetectable in the final follow-up samples. The m*MGMT* profile in sEV-DNA was observed with a MRI image compatible with a partial response in the first follow up and complete response after treatment. Consequently, the mMGMT levels of patient ID1 showed consistency with the imaging data, suggesting that methylation levels may underpin the results obtained through MRI (Fig. 4B). Patient ID28 had a similar course to patient ID1. However, in this case, it was not possible to obtain further follow-up samples to confirm a favorable evolution, with a PFS of 32 months.

Patients ID2 and ID32 also carried an *MGMT* methylated tumor sample. m*MGMT* was also detected in sEV-DNA in all blood samples obtained during the follow-up. Both patients progressed without significantly decreasing methylation levels in their blood. Specifically, patient ID2 was diagnosed with *MGMT* methylated GB and underwent tumor resection. Postoperative MRI revealed remaining tumor tissue and baseline sEV-DNA showed m*MGMT*. Although there was no evidence of disease progression on the first follow-up MRI scan, the monitoring sample revealed that m*MGMT* ∆Ct was increased. After 3 months, MRI showed disease progression, suggesting that m*MGMT* ∆Ct elevation could predict tumor recurrence. The patient started a new treatment which slightly decreased the ∆Ct values, but eventually the patient progressed and died (Fig. 4C). Patient ID32 had a similar situation to the previous patient. Therefore, the dynamics of m*MGMT* in sEV-DNA were closely associated with the MRI results and patient prognoses, providing a valuable tool for the follow-up of patients with m*MGMT.*

Patient ID34 underwent complete resection of a methylated *MGMT* tumor. The methylation remained at the baseline sEV-DNA sample, but was no longer observed in the remaining follow-up. MRI performed one month before sample collection showed no progression; however, the patient progressed and died two months after the sample was obtained; therefore, it was not possible to obtain a second follow-up at that stage to corroborate the m*MGMT* status.

Patients ID12 and ID41 had an absence of m*MGMT* in the tumor and, as expected, in all sEV-DNA samples obtained. In the case of patient ID40, who underwent complete resection of an unmethylated *MGMT* GB tumor, sEV-DNA methylation in the baseline sample was detected. In the first follow-up sample, the patient no longer showed mMGMT and remained stable until 9 months.

## Discussion

Cancer management involves challenges associated with diagnosis, prognosis, and prediction of treatment response. m*MGMT* is a well-known predictive biomarker in patients with GB^4,7,31^, and its assessment is an important factor in choosing the best treatment strategy or selecting patients for clinical trials. Its reliable assessment in liquid biopsy would therefore be a robust tool of great value in the clinical follow-up of GB patients^24^.

Liquid biopsy results are highly dependent on tumor type, stage and location and can achieve sensitivities of over 90% in metastatic patients^32^. However, in contrast to other solid tumors, the procedure of conducting liquid biopsies relying on ctDNA in glioma patients presents notable challenges due to the persistently low levels of detectable biomarkers in their bloodstream. Achieving the highest sensitivities typically involves assays utilizing cerebrospinal fluid (CSF); however, these methods necessitate invasive sampling^33,34^. Initially, these challenges were attributed to the presence of the blood–brain barrier (BBB). Nonetheless, recent studies have shed light on disruptions in the BBB in glioma patients, complicating our understanding of this limitation^35^. On the other hand, García-Romero et al. have provided evidence that sEVs can effectively cross the intact BBB and be detected in peripheral blood. This discovery overcomes the previous limitation and introduces a new way to perform liquid biopsy in glioma patients, potentially improving diagnostic accuracy and clinical management strategies^16^.

The field of EVs has not been without its challenges, requiring the development and refinement of methodologies for EV isolation and characterization. This diversity of methodologies poses significant hurdles to standardization efforts^36^. As emphasized by the International Society of Extracellular Vesicles, it is imperative to use at least two different and complementary techniques for sample characterization, such as electron microscopy, nanoparticle tracking analysis or protein marker detection. In our study, we chose the first two methods^19^. The comprehensive characterization obtained using these techniques confirms the successful isolation of EVs. While we acknowledge the potential presence of platelets or lipoproteins, we maintain that this inherent limitation does not compromise the integrity of our results. Our focus on *mMGMT*, which is exclusively associated with oncological processes, remains unaffected by these considerations.

To date, studies to determine m*MGMT* status in blood-based liquid biopsy using ctDNA in patients with gliomas obtained a sensitivity ranging from 11 to 76.6%^10,13–15,37–41^, achieving the best results when the blood sample was obtained prior to surgical resection. For example, the methylation status of *MGMT* and other genes was investigated in a cohort of 28 glioblastoma patients treated with 1,3-bis(2-chloroethyl)-1-nitrosourea or temozolomide plus cisplatin, and compared to tissue, the sensitivity for detecting *MGMT* methylation in serum was 62.5%, with a specificity of 92.3%^41^. Other study showed that the methylation status of the *MGMT* promoter in serum had a sensitivity of 66.7% and a specificity of 100.0%^40^. Similarly, Lavon et al. reported a sensitivity of 59% and a specificity of 100% for *MGMT* methylation^38^. Notably, the study by Gong et al. showed the best performance with a sensitivity of 76.61% and a specificity of 98.28%^15^. Utilizing an alternative approach focused on analyzing RNA content within EVs, Mut, Melike et al. developed a LASSO-penalized binomial regression model. This model, employing 17 out of 569 differentially expressed genes as predictors in EVs, achieved a predictive accuracy of 91% sensitivity and 73% specificity in determining the *MGMT* methylation status^42^. Additionally, it is noteworthy to mention that no studies evaluating the methylation of *MGMT* using DNA in EVs have been identified to date. Therefore, our study provides an unprecedented increase in the percentage of marker agreement between tissue and plasma, reaching 90% concordance with a sensitivity of 87.5% and a specificity of 92% in subtotal resection cases. Furthermore, given the primary objective of a predictive biomarker is forecast treatment response, our results suggest that *MGMT* methylation in tumor approaches statistical significance in predicting survival although our limited sample size. Additionally, patients undergoing subtotal resection may also benefit from the *mMGMT* biomarker in EVs in terms of survival, albeit without reaching statistical significance. While acknowledging the limitations of the limited sample size, our data suggest that *mMGMT* in EVs may have potential significance as a predictive biomarker in glioblastoma. Therefore, further investigation is needed to support our observations and facilitate the integration of the *mMGMT* biomarker into clinical contexts.

We observed that lower concordance in *MGMT* methylation between tumor and sEV was associated with patients who underwent complete resection. This suggests that detecting *MGMT* methylation in sEV is more challenging in cases where the tumor has been previously removed. Thus, considering our results in tumors with incomplete resection and the results obtained in monitored patients, our study showed promising results, achieving the highest sensitivity reported to date for sEV. Therefore, we speculate that if preoperative blood samples had been available in our study, the sensitivity achieved in sEV DNA analysis would likely have been further increased. This speculation is supported by the established sensitivity of preoperative samples demonstrated by previous liquid biopsy research^15,39^. The potential increase in sensitivity underscores the critical role of timing of sample collection and highlights the importance of optimizing sample collection protocols to increase the potential utility of liquid biopsy approaches.

To our knowledge, only one study has previously used sEV-DNA-based liquid biopsy to investigate *IDH* status in patients with GB^16^. Alternatively, two studies have compared sEV-DNA-based liquid biopsy with cDNA-based liquid biopsy in paired samples. The study by Allenson et al. detected a higher rate of *KRAS* mutations in sEV-DNA compared with ctDNA with sensitivities of 66.7%, 80% and 85% of patients with localized, locally advanced and metastatic pancreatic cancer, respectively, whereas the results obtained in ctDNA were 45.5%, 30.8% and 57.9%, respectively^43^. Bernard et al. achieved similar results, obtaining higher sensitivity using sEV-DNA than ctDNA^44^. Furthermore, serum analysis has revealed that EGFRvIII can be detected non-invasively in EV, with an overall clinical sensitivity of 81.58% and specificity of 79.31%^45^. sEV also contain abundant miRNAs and proteins associated with proliferation, angiogenesis, cell migration, immune response and histone modification, which could be used as biomarkers in this disease^46,47^. Nevertheless, each of these studies employed varying sample types (serum/plasma), DNA isolation methodologies, and techniques for mMGMT determination. Consequently, it is paramount to exercise caution when interpreting and comparing absolute values, recognizing the inherent limitations associated with these methodological disparities.

Reaching 100% sensitivity in liquid biopsy tests is difficult due to the low concentration of tumor markers in the pool of molecules in the blood. However, this same condition makes the lack of specificity an uncommon event. In our study, we found two patients in whom *MGMT* promoter was methylated in sEV-DNA but was not detected in tumor tissue DNA (ID14 and ID40). Since false positives in liquid biopsy are rare, it leads us to suspect that it might have been a false negative in the tumor tissue determination mainly due to tumor heterogeneity, one of the main limitations of tissue sampling, given that it is difficult for a single sample to represent the entire lesion^8,32,48^.

The role that liquid biopsy can play in the monitoring of patients with GB is not well established to date, given that the results published on this disease have been very limited. In a subgroup of 12 patients, Bagley et al. reported that ctDNA levels obtained prior to initial tumor resection in adult patients with newly diagnosed GB increased with progression and remained stable in patients who did not progress^49^, data that were corroborated by Fontanilles et al.^50^. Muralidharan et al. monitored *TERT* mutations in ctDNA in five patients, concluding that their frequency reflects the clinical course of the disease with levels that decrease after surgery and increase with tumor progression^51^. In our study, we recruited twenty samples from eight patients with baseline and at least one follow-up sample. Five of these patients had m*MGMT* GB. We found that patients with *mMGMT* GB harboring detectable m*MGMT* at baseline and during follow-up in blood samples were patients who showed tumor progression. However, patients with m*MGMT* in sEV-DNA in the baseline sample that decreased in the follow-up samples were patients with radiographic evidence of tumor response. Therefore, changes in ∆Ct levels in sEV-DNA provide relevant predictive information to manage and monitor the outcome of patients with GB.

Methylation in sEV DNA in cancer patients has only been detected in the study by Zavridou et al., who detected methylation in the *GSTP1* and *RASSF1A* genes^52^. To our knowledge, our study shows for the first time that detection of m*MGMT* in sEV-DNA-based liquid biopsy in patients with GB is feasible and greatly improves on the previously published sensitivity calculated using circulating blood DNA. These results are supported by a recent study conducted on GB cell culture-derived sEVs in a genome-wide methylation profiling approach. In this study, Maire et al. were able to correctly categorize tumors according to the Heidelberg classification, including the *MGMT* promoter methylation status^53^. Thus, the in vitro study conducted by Maire et al. together with our in vivo data provide the basis for the diagnostic characterization of genome-wide methylation in sEV-DNA, which would allow the molecular classification and disease monitoring of CNS tumors in patients in whom tumor samples are not available.

## Conclusions

We report for the first time the detection of m*MGMT* in sEV-DNA with a sensitivity and specificity of 87.5% and 90%, respectively. The presence of MGMT in sEV-DNA appeared to show an association with patient OS and facilitated patient monitoring; however, statistical power limitations prevent a definitive determination. Therefore, the results obtained here represent an important contribution to the field of extracellular vesicle-based liquid biopsy, suggesting that it reflects the heterogeneity of tumor tissue and represents a promising tool for biomarker detection. However, further larger studies are required to confirm our findings.

## Supplementary Information

Supplementary Figure 1.

Supplementary Figure 2.

## Data Availability

The datasets used and/or analyzed during the current study are available from the corresponding author on reasonable request.

## Abbreviations

CNS: Central nervous system
WHO: World Health Organization
IDH: Isocitrate dehydrogenase
GB: Glioblastoma
TMZ: Temozolomide
OS: Overall survival
MGMT: O6-methylguanine-DNA methyltransferase
mMGMT: Methylated MGMT
ctDNA: Circulating tumor DNA
sEVs: Extracellular vesicles
BBB: Blood–brain barrier
sEV-DNA: DNA extracted from sEVs
ECOG: Eastern Cooperative Oncology Group
UC: Ultracentrifugation
PBS: Phosphate buffered saline
NTA: Nanoparticle tracking analysis
TEM: Transmission electron microscopy
TE 1×: Tris-EDTA buffer solution
PPV: Positive predictive value
NPV: Negative predictive value
95% CI: 95% Confidence intervals
DP: Disease progression
MRI: Magnetic resonance imaging
