# Supplementary material for: Evaluation of the clinical use of MGMT methylation in extracellular vesicle-based liquid biopsy as a tool for glioblastoma patient management
Source: Sci Rep. 2024 May 18;14:11398. doi: 10.1038/s41598-024-62061-8 (PMC11102540; doi:10.1038/s41598-024-62061-8)
Supplement: Supplementary file 1 — Supplementary Figure 1. [file 41598_2024_62061_MOESM1_ESM.pdf]

Supplementary Figure 1.
Results of MGMT methylation in healthy donors

Patient ID	*MGMT* sEV-DNA methylation (Baseline)
C1	U (0.0%)
C2	U (0.0%)
C3	U (0.0%)
C4	U (0.0%)
C5	U (0.0%)
C6	U (0.0%)
C7	U (0.0%)
C8	U (0.0%)

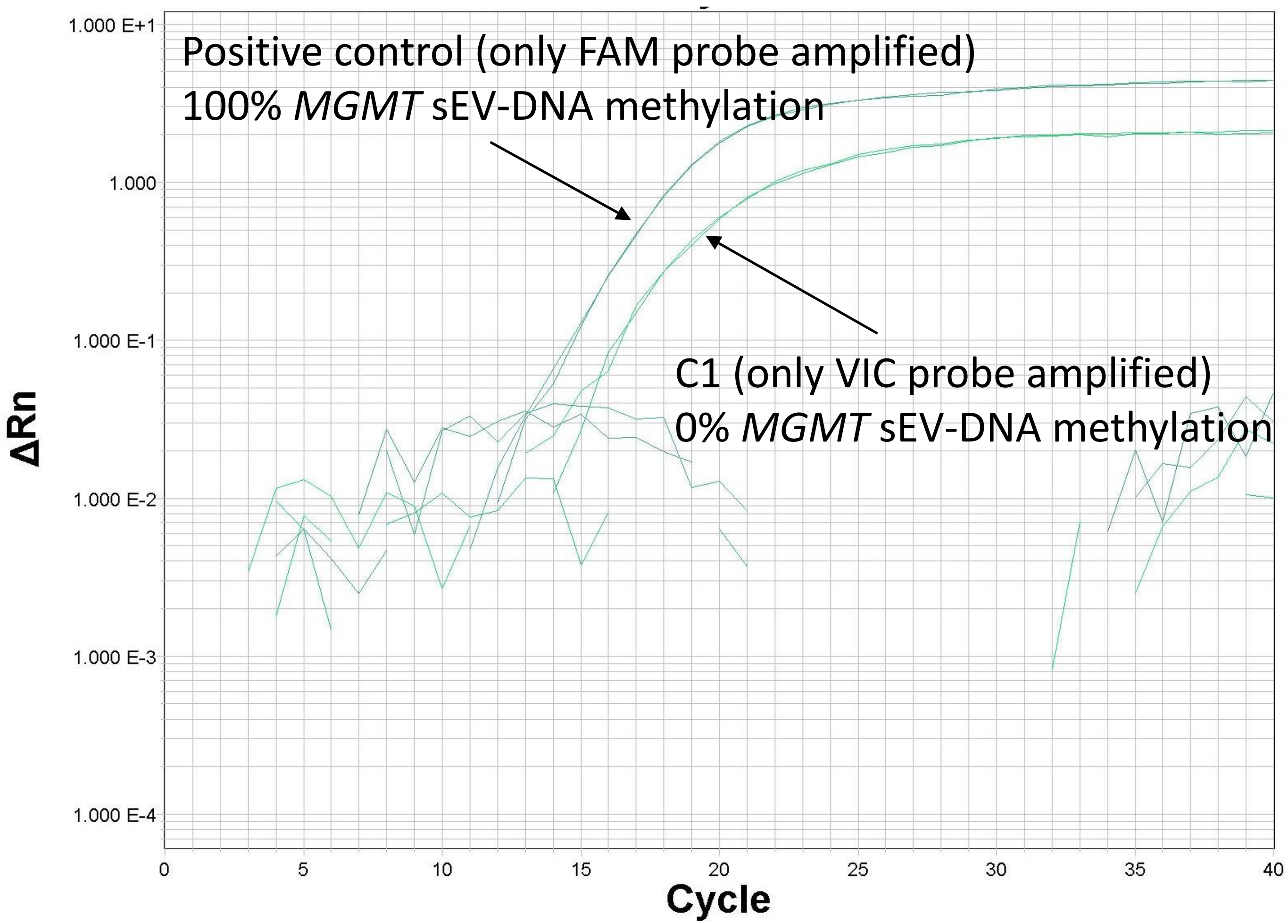

None of the healthy donors showed MGMT methylation in the samples tested. An example of the results obtained for control sample 1 (C1) is shown.
