# Supplementary material for: Evaluation of the clinical use of MGMT methylation in extracellular vesicle-based liquid biopsy as a tool for glioblastoma patient management
Source: Sci Rep. 2024 May 18;14:11398. doi: 10.1038/s41598-024-62061-8 (PMC11102540; doi:10.1038/s41598-024-62061-8)
Supplement: Supplementary file 2 — Supplementary Figure 2. [file 41598_2024_62061_MOESM2_ESM.pdf]

Supplementary Figure 2.
PCR primers used in this paper

Primer	Sequence (5'-> 3')	Size (bp)
MGMT nested PCR	F: GGATATGTTGGGATAGTT<br>R: CCTACAAAACCACTCRAAACT	166 bp
MGMT methylated qMSP	F: TTTCGACGTTCTAGGTTTTCGC<br>R: GCACTCTTCCGAAAACGAAACG<br>Taqman Probe: FAM-CAAATCGCAAACGATA-MGB-NFQ	81 bp
MGMT unmethylated qMSP	F: TTTGTGTTTTGATGTTTGTAGGTTTTTGT<br>R: AACTCCACACTCTTCCAAAAACAAAACA<br>Taqman Probe: VIC-CAAATCACAAACAATA-MGB-NFQ	93 bp
